# Functional lung avoidance for individualized radiotherapy (FLAIR): study protocol for a randomized, double-blind clinical trial

**DOI:** 10.1186/1471-2407-14-934

**Published:** 2014-12-11

**Authors:** Douglas A Hoover, Dante PI Capaldi, Khadija Sheikh, David A Palma, George B Rodrigues, A Rashid Dar, Edward Yu, Brian Dingle, Mark Landis, Walter Kocha, Michael Sanatani, Mark Vincent, Jawaid Younus, Sara Kuruvilla, Stewart Gaede, Grace Parraga, Brian P Yaremko

**Affiliations:** Department of Radiation Oncology, London Regional Cancer Program, 790 Commissioners Rd. E, London, Ontario N6A 4L6 Canada; Department of Oncology, Western University, London, Ontario Canada; Department of Medical Biophysics, Western University, London, Ontario Canada; Imaging Research Laboratories, Robarts Research Institute, 1151 Richmond Street, London, Ontario N6A 5B7 Canada; Department of Medical Imaging, Western University, London, Ontario Canada

**Keywords:** Functional imaging, Radiotherapy, Non-small cell lung cancer, Helium-3 MRI, Quality of life

## Abstract

**Background:**

Although radiotherapy is a key component of curative-intent treatment for locally advanced, unresectable non-small cell lung cancer (NSCLC), it can be associated with substantial pulmonary toxicity in some patients. Current radiotherapy planning techniques aim to minimize the radiation dose to the lungs, without accounting for regional variations in lung function. Many patients, particularly smokers, can have substantial regional differences in pulmonary ventilation patterns, and it has been hypothesized that preferential avoidance of functional lung during radiotherapy may reduce toxicity. Although several investigators have shown that functional lung can be identified using advanced imaging techniques and/or demonstrated the feasibility and theoretical advantages of avoiding functional lung during radiotherapy, to our knowledge this premise has never been tested via a prospective randomized clinical trial.

**Methods/Design:**

Eligible patients will have Stage III NSCLC with intent to receive concurrent chemoradiotherapy (CRT). Every patient will undergo a pre-treatment functional lung imaging study using hyperpolarized ^3^He MRI in order to identify the spatial distribution of normally-ventilated lung. Before randomization, two clinically-approved radiotherapy plans will be devised for all patients on trial, termed *standard* and *avoidance*. The standard plan will be designed without reference to the functional state of the lung, while the avoidance plan will be optimized such that dose to functional lung is as low as reasonably achievable. Patients will then be randomized in a 1:1 ratio to receive either the standard or the avoidance plan, with both the physician and the patient blinded to the randomization results. This study aims to accrue a total of 64 patients within two years. The primary endpoint will be a pulmonary quality of life (QOL) assessment at 3 months post-treatment, measured using the functional assessment of cancer therapy–lung cancer subscale. Secondary endpoints include: pulmonary QOL at other time-points, provider-reported toxicity, overall survival, progression-free survival, and quality-adjusted survival.

**Discussion:**

This randomized, double-blind trial will comprehensively assess the impact of functional lung avoidance on pulmonary toxicity and quality of life in patients receiving concurrent CRT for locally advanced NSCLC.

**Trial registration:**

Clinicaltrials.gov identifier: NCT02002052.

## Background

### Radiation induced lung injury

Lung cancer is the most common cause of cancer death in men and women worldwide [1]. The large majority of lung cancer patients present with non-small cell lung cancer (NSCLC), and of these, approximately 30% present with locally advanced (stage III) disease. The current standard of care for locally advanced unresectable NSCLC is concurrent chemotherapy (CRT) with curative intent [2, 3]. Survival improvements of concurrent CRT over sequential CRT have been well-defined after multiple randomized trials, with concurrent CRT conferring a 10% overall survival benefit at two years [4, 5]; however, such treatment is associated with an increased risk of radiation-induced lung injury (RILI), including radiation pneumonitis (RP).

Clinically symptomatic RP occurs in 30-40% of patients after concurrent CRT and can have a major impact on quality of life, sometimes resulting in oxygen dependence, and in severe cases is fatal [6, 7]. Several factors are currently used to attempt to predict RP and to mitigate risk. Most of these predictive factors are metrics of the radiation dose delivered to normal lung, such as the volume of lung receiving ≥20 Gy of radiation, the mean lung dose and the dose per fraction of radiation. For example, a recent meta-analysis found that the volume of lung receiving at least 20 Gy (V20) is the best individual predictor of RP risk; a V20 > 40% is associated with a 35% risk of symptomatic RP, and >3% risk of fatal RP [7], supporting several previous single-institution studies [8] and a systematic review [6].

The risk of RP limits the radiotherapy dose that can be safely delivered. Although numerous modelling studies have indicated that higher doses of radiotherapy should be associated with improved oncologic outcomes, randomized data have shown that dose escalation leads to excess lung toxicity. The recent landmark RTOG 0617 randomized trial compared standard vs. high dose radiotherapy (60 Gy vs. 74 Gy), with concurrent chemotherapy, for locally advanced NSCLC. Overall survival at 18-months was 66.9% in the 60-Gy arm and 53.9% in the 74-Gy arm (p < 0.001), indicating inferior survival with dose-escalation [9].

Toxicity outcomes from RTOG 0617, as scored by the health-care providers, did not initially appear to explain the inferior survival in the high-dose arm. Although there were more deaths due to radiation pneumonitis in the high-dose arm (5% vs. 1%) this did not meet statistical significance and only accounted for a small proportion of the overall survival difference between the two arms. However, *patient-reported outcomes* indicated a different toxicity profile; respiratory toxicity was common and was not often detected by the health-care providers. In the high-dose arm, 49% of patients exhibited a clinically-meaningful decline in the pulmonary quality of life (QOL) at 3-months, compared to 31% of patients in the low-dose arm (p = 0.024). Pulmonary QOL was also an important survival metric overall. Baseline QOL predicted for overall survival (OS) in multivariable analysis, more so than stage, performance status and other conventional prognostic factors [9].

In summary, for patients treated with standard concurrent CRT for locally advanced lung cancer, RP is a major source of morbidity, impairs quality of life, and can result in treatment-related death. RP also limits the dose of radiotherapy that can be safely delivered, and currently precludes radiotherapy dose escalation. RP is not well-ascertained by healthcare providers; in contrast, patient-reported QOL outcomes appear to be a powerful tool to capture pulmonary toxicity outcomes [9]. Clearly, better methods are needed to reduce pulmonary toxicity for patients undergoing concurrent CRT for lung cancer.

### Functional lung avoidance

At present, radiation treatment planning for advanced lung cancer is based upon minimizing radiation dose to the total lung, regardless of the degree of function at any particular point within that lung. This approach does not account for the fact that lung tissue can be heterogeneous, especially in smokers, whose lungs are frequently characterized by large regions of unventilated parenchyma such as bullae. Ideally, radiotherapy treatment planning should be able to exploit these regional differences in lung function by minimising dose to the more highly functional lung while favouring radiation deposition in areas of less highly-functioning or non-functioning lung.

Over the last decade, functional measurements and maps obtained from thoracic imaging have been evaluated for use in lung cancer radiation therapy planning with single photon emission computed tomography (SPECT) [10, 11], high resolution four-dimensional x-ray computed tomography (4DCT) [12, 13], and hyperpolarized noble gas magnetic resonance imaging (MRI) [14, 15]. All of these techniques potentially facilitate the delineation of regional pulmonary function for lung cancer radiation treatment planning, resulting in reduced radiation dose to well-functioning lung without dose decreases to the treatment target volume [11, 15, 16]. However, it is not clear which of these is optimum, as each has its own merits and drawbacks. For example, one of the most widely studied techniques is SPECT. Although the incorporation of SPECT for lung cancer radiation therapy planning has been promising, there are some inherent limitations that may preclude its routine clinical use, mainly related to image artefacts stemming from radiolabelled tracers depositing in the major airways [17], requiring significant post-processing to remove, and sometimes resulting in distortion of the underlying ventilation signal.

### Hyperpolarized noble gas MRI

Hyperpolarized ^3^He MRI provides an alternative to ventilation SPECT [15, 18]. ^3^He MRI provides relatively high spatial and temporal resolution of respiratory function, can be used safely in a wide variety of respiratory patients and does not release ionizing radiation [19]. Although ^3^He MRI has several inherent advantages, it will not likely achieve widespread clinical use due to cost and a limited global supply of ^3^He gas for research purposes. Several alternative imaging techniques appear promising and are expected to be available for widespread clinical use in the future, including ^129^Xe MRI, which is currently less well-developed than ^3^He MRI [20], ^1^H Fourier decomposition methods [21], and 4DCT-based ventilation mapping [22]. If the benefits of functional lung avoidance can be demonstrated now using ^3^He MRI, then other, more easily accessible ventilation imaging modalities (e.g. 4DCT and ^129^Xe MRI) may allow for more widespread implementation of functional lung avoidance radiotherapy in future.

## Methods/design

### Objectives

#### General objective

To determine if functional lung avoidance based on ^3^He MRI improves quality of life outcomes for patients with NSCLC undergoing concurrent CRT.

#### Primary endpoint

- Pulmonary QOL 3-months post-treatment

○ Measured using the Functional Assessment of Cancer Therapy—Lung Cancer Subscale (FACT-LCS)

#### Secondary endpoints

- Pulmonary QOL at other time-points

○ Measured using the FACT-LCS

- Other QOL scores

○ FACT—Trial Outcomes Index (FACT-TOI)

○ FACT—Lung (FACT-L) and subscales

- Provider-reported toxicity (including RP and esophagitis)

○ Assessed by the National Cancer Institute Common Toxicity Criteria (NCI-CTC) version 4

- Overall Survival

○ Defined as time from randomization to death from any cause

- Progression-free survival

○ Time from randomization to disease progression at any site or death

○ Progression defined according to RECIST 1.1

- Quality-Adjusted Survival (based on EQ-5D)

### Study design

**Figure 1:**
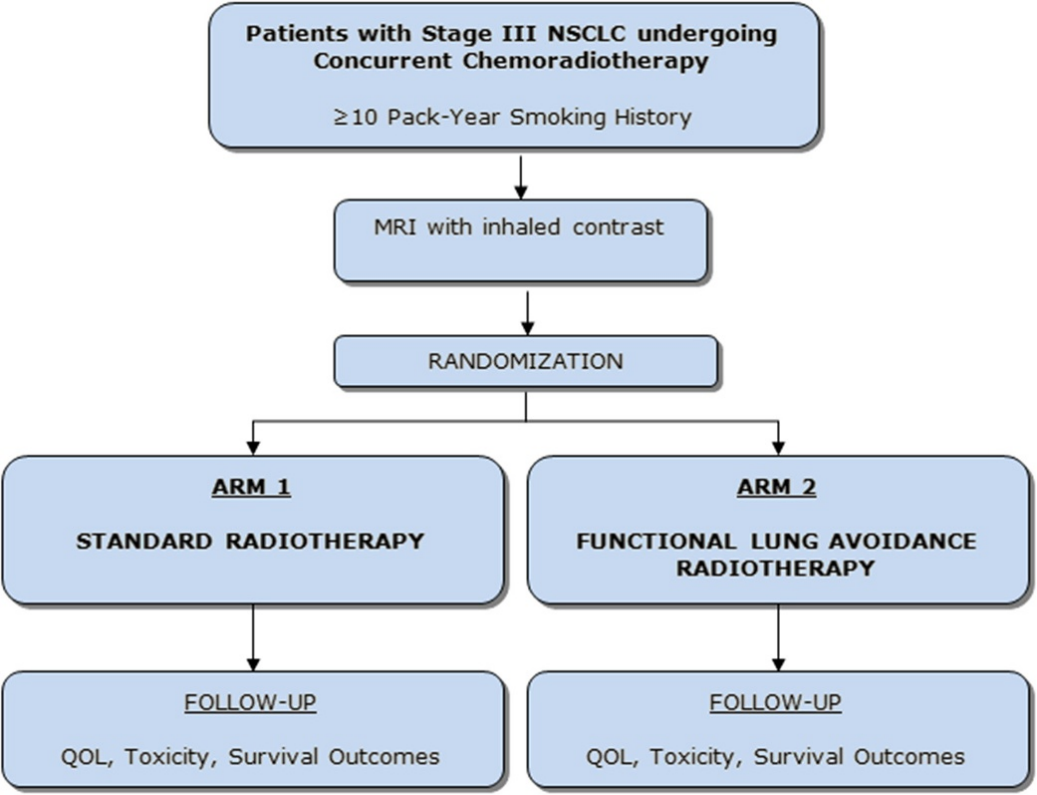
**Study design: patients will be randomized in a 1:1 ratio between Arm 1 (standard radiotherapy) and Arm 2 (functional lung avoidance radiotherapy).**

This study is a double-blinded randomized controlled trial (Figure 1).

### Patient selection

#### Inclusion criteria

- Age 18 or older
- Willing to provide informed consent
- ECOG performance status 0-2
- Histologically confirmed non-small cell lung carcinoma
- Locally advanced Stage IIIA or IIIB lung carcinoma according to AJCC 7^th^ edition
- History of at least 10-pack-years of smoking
- Ambulatory and able to perform the Six Minute Walk Test (6MWT)
- FEV_1_ ≥ 750 ml or ≥30% predicted
- Not undergoing surgical resection
- Assessment by medical oncologist and radiation oncologist, with adequate bone marrow, hepatic and renal function for administration of platinum-based chemotherapy, as determined by the treating physicians

#### Exclusion criteria

- Subject has an implanted mechanically, electrically or magnetically activated device or any metal in their body which cannot be removed, including but not limited to pacemakers, neurostimulators, biostimulators, implanted insulin pumps, aneurysm clips, bioprosthesis, artificial limb, metallic fragment or foreign body, shunt, surgical staples (including clips or metallic sutures and/or ear implants)
- In the investigator’s opinion, subject suffers from any physical, psychological or other condition(s) that might prevent performance of the MRI, such as severe claustrophobia.
- Serious medical comorbidities (such as unstable angina, sepsis) or other contraindications to radiotherapy or chemotherapy
- Prior history of lung cancer within 5 years
- Prior thoracic radiation at any time
- Metastatic disease. Patients who present with oligometastatic disease where all metastases have been ablated (with surgery or radiotherapy) are candidates if they are receiving concurrent CRT to the thoracic disease with curative intent
- Inability to attend full course of radiotherapy or follow-up visits
- Pregnant or lactating women

### Pre-treatment evaluation

- History and physical examination by a radiation oncologist and medical oncologist within 12 weeks prior to enrolment onto study
- Histological confirmation of non-small cell carcinoma
- Standard staging within 12 weeks prior to initiation of chemotherapy including:

○ CT chest and upper abdomen

○ Whole body FDG-PET-CT scan (currently funded for stage III NSCLC in Ontario)

○ CT head or MRI head

- Pulmonary function tests within 12 weeks of initiation of radiotherapy showing adequate FEV_1_: the best value obtained pre- or post-bronchodilator must be ≥750 ml or ≥30% predicted
- Bloodwork: CBC with differential, Hemoglobin, AST, ALT, bilirubin, creatinine should be done before 1^st^ cycle of chemotherapy. If any tests are missed they must be done prior to start of radiation.
- Pregnancy test for women of child-bearing age

### Study visits

Subjects will visit the research center three times: pre-treatment, three months post-treatment, and 12 months post-treatment. ^3^He MRI and non-contrast chest CT will be performed on the first visit only. Subjects will undergo pulmonary function tests, Forced Oscillation Technique, 6MWT, and QOL questionnaires at each visit.

#### Pulmonary function tests

Full pulmonary function tests including spirometry, plethysmography and diffusing capacity of carbon monoxide (DL_CO_) will be performed according to the joint American Thoracic Society/European Respiratory Society (ATS/ERS) guidelines [23–27] using the MedGraphics (Elite Series, MedGraphics Corporation, St. Paul, MN USA) whole-body plethysmograph and/or ndd EasyOne Spirometer (ndd Medical Technologies Inc., Andover, MA USA).

Airwave oscillometry will be performed using the TremoFlo™ (THORASYS Thoracic Medical Systems, Halifax, NS). Airwave oscillometry measures the mechanics of the respiratory system and evaluates lung function without patient effort by superimposing a gentle multi-frequency airwave onto the patient’s respiratory airflow. Patients breathe normally throughout the measurement sequence for less than a minute via a disposable mouthpiece.

#### Six minute walk test

Subjects will perform the 6MWT according to ATS guidelines [28]. Subjects will rate their dyspnea and overall fatigue at baseline and at the end of the exercise using the Borg Scale [29].

#### CT

Low dose, thoracic multi-detector row computed tomography will be performed with the same breath-hold volume and maneuver used for MRI. CT imaging will be performed using a 64-slice (General Electric Health Care, Milwaukee) scanner. In order to match CT and MRI breath-hold volumes and anatomy, subjects will be scanned in the supine position during inspiration breath-hold from functional residual capacity (FRC) after inhaling one litre of N_2_ gas as previously described [30].

#### MRI

MR imaging will be performed using a 3.0 T MR750 system (GE Health Care, Milwaukee, Wisconsin) using a whole-body gradient amplitude of 1.94 G/cm and a single-channel, rigid elliptical transmit/receive chest coil (Rapid Biomedical GmbH, Wuerzburg, Germany). For ^1^H and ^3^He MRI, subjects will be instructed to inhale from FRC a gas mixture from a one-litre Tedlar bag (Jensen Inert Products, Coral Springs, FL). Image acquisition will be performed during a 16-second breath hold. Coronal (anatomical) ^1^H MRI will be performed using the whole-body radiofrequency coil and ^1^H fast-spoiled, gradient-recalled echo sequence using a partial echo (16 s total data acquisition, repetition time [TR] =4.7 ms, echo time [TE] =1.2 ms, flip angle =30°, field of view =40 cm, bandwidth =24.4 kHz, matrix =128 × 80, 15-17 slices, 15 mm slice thickness). ^3^He MRI static ventilation images will be acquired using a fast-gradient echo method using a partial echo (14 s total data acquisition, TR/TE/flip angle =4.3 ms/1.4 ms/7°, field of view =40 cm, bandwidth =48.8 kHz, matrix =128 × 80, 15-17 slices, 15 mm slice thickness) [30]. A pulse oximeter lead will be attached to all subjects to monitor their heart rate and oxygen saturation. All subjects will have supplemental oxygen provided via nasal cannula at a flow rate of two litres per minute during the scanning process.

Adverse events and pulse oximetry measurements during MRI will be recorded. If oxygen saturation falls to <80% continuously for ≥15 seconds, scanning will be discontinued and the patient will be provided supplemental oxygen, as necessary, until oxygen saturation recovers to the patient’s baseline value. The patient will then be discontinued from the study. Oxygen desaturation below 88% during a ^3^He/^129^Xe breath-hold will be considered an adverse event.

### Radiotherapy

#### Technique

Patients will be treated with intensity modulated radiotherapy (IMRT). Volumetric modulated arc therapy (VMAT) is preferred, but IMRT can be delivered using static-beam techniques or other rotational techniques (*e.g.* Tomotherapy™). For rotational techniques, care must be taken to minimize dose to the contralateral lung. For each patient, both plans (standard and functional-lung-avoidance) must be planned using the same delivery technique. Respiratory gating is allowed for tumours with >7 mm of respiratory motion.

#### Immobilization and localization

All patients will be positioned with arms over their heads, chin extended and immobilized according to institutional standards. Patients will undergo a planning 4DCT simulation encompassing the entire lung volume, typically extending from level of the C5 to L1 (below diaphragm), with 3 mm slice thickness. Intravenous contrast may be used to improve delineation of target volumes when the target is centrally located, at the discretion of the treating radiation oncologist. The planning CT may be fused with other available standard diagnostic imaging (MRI, CT or PET).

#### Functional lung delineation on planning CT

Pulmonary segmentation of ventilatory patterns will be performed using semi-automated methods, as previously described [22]. The ^3^He MRI containing the delineated areas of functional lung will be fused to the breath-hold CT and the planning CT. Due to differences in tidal volume between the two scans, deformable registration will be required for most cases. To ensure accuracy, the fusion will be inspected by a physicist and the treating physician.

#### Radiotherapy volume definitions

The radiotherapy planning and delivery parameters used in this study are based on current consensus guidelines for treatment of locally advanced lung cancer. The gross tumour volume (GTV) is defined as the visible tumour and involved lymph nodes based on CT or PET imaging (nodes must be 1 cm or more in short axis or necrotic on CT, or PET positive, or biopsy-proven to contain carcinoma). Elective nodal irradiation will not be used. Nodes that are <1 cm and PET-negative may not be included in the GTV unless they are necrotic-appearing.

Radiotherapy may be delivered using either a free-breathing or a gated technique. For the free-breathing treatment, a GTV will be delineated at end-inspiration and end-expiration. At the discretion of the radiation oncologist, the GTV may also be delineated on other phases of the breathing cycle (e.g. in cases involving significant hysteresis). All contoured GTVs shall then be fused to create the internal GTV (IGTV). For patients treated with respiratory gating, a subset average CT will be created by averaging several phases around end-expiration such that tumour motion is minimized while maintaining a clinically-acceptable gating window (typically, the 40-60% phases will be used for the subset average). The GTV will be contoured on the subset average scan and treatment will be delivered within the defined gating window. The GTV may also be contoured on the end-inspiration phase to aid in image-guidance using free-breathing cone-beam CT.

For all patients, a 5 mm margin will be added for microscopic disease to create an Internal Target Volume (ITV). This margin may be decreased at natural boundaries to microscopic extension (e.g. bone), or increased up to 8 mm in areas of uncertainty. For the planning target volume (PTV), a further 5 mm expansion will be added to the ITV in all directions.

For the purposes of radiotherapy planning, two ventilation regions will be created representing different levels of lung ventilation. The structure lung-vent will represent lung with any measurable ventilation. The structure lung-avoid will represent lung tissue that has normal ventilation according to previously published methodology [23]. The structure lung-avoid will be preferentially spared. Due to registration errors and various imaging artefacts, portions of the lung ventilation maps are expected to be outside the anatomical lung boundary of the planning CT. For this reason, lung ventilation structures will be cropped so that they are contained within the anatomically-defined lungs.

#### Prescription and dose constraints

The prescription dose will be 60 Gy in 30 fractions, with 95% of the PTV to receive 95% of the prescribed dose. Target dose constraints were adapted from the RTOG 0617 study protocol (Table 1) [31]. Normal structures including spinal cord, right lung, left lung, oesophagus and heart should be contoured on each CT slice of the planning CT. The lung volume at risk is defined as the total lung minus IGTV. The oesophagus should be contoured from the caudal aspect of the cricoid to the gastroesophageal junction. The heart contours should extend from the beginning of the ascending aorta down to the apex of the heart.

**Table 1:** **Normal tissue dose constraints for radiotherapy planning**

Structure	Dose constraints for 60 Gy in 30 fractions
Spinal Cord	Max dose <50 Gy
Lungs	V20 < 37%
	V05 < 90%
	Mean dose <21 Gy
Oesophagus	Mean dose <34 Gy
	Minimize V60
Heart	60 Gy to <1/3
	45 Gy to <2/3
	40 Gy to <3/3

The spinal cord dose constraint cannot be exceeded for any reason. It is strongly recommended that the other dose constraints not be exceeded. If any dose constraint needs to be exceeded in order to achieve adequate coverage of the PTV, approval by the treating physician is required.

#### Planning workflow and blinding

Before randomization, each patient will require two clinically-approved treatment plans meeting the constraints defined above. One plan will be designed without reference to the functional status of the lung (termed *standard plan*). The second plan (termed *avoidance plan*) will be optimized such that dose to functional lung is as low as reasonably achievable, with an aim to minimize the V5, V20, and mean dose within the functional lung. For a given patient, both standard and avoidance plans must use the same treatment technique, (i.e. VMAT, static-gantry IMRT or Tomotherapy™). While this protocol does allow for static-beam IMRT, a rotational technique is preferred.

The standard plan will always be completed first, followed by the avoidance plan. Avoidance plans will, in general, be more heterogeneous than standard plans. While homogeneous plans may be more aesthetically pleasing, there is no evidence to suggest that they are superior. Thus, PTV homogeneity constraints and conformity constraints will be relaxed for avoidance plans. Nonetheless, hotspots of 105% or greater will be avoided outside the PTV.

The structure lung-avoid (representing normally-ventilated lung) will be preferentially avoided in order to devise the avoidance plan. In general, the anatomically-defined lungs should not be used during optimization of the avoidance plan; rather, the structure lung-vent should be used in its place. If necessary, a maximum V20 constraint for the anatomical lungs may be used which is the smaller of: 37%, or 3% greater than the V20 generated for the anatomical lungs in the standard plan. Compared with the standard plan, the goals for the avoidance plan will be as follows:

- 3% reduction in the V20 for lung-avoid, and/or a 1.5% reduction in V20 for lung-vent
- 1.5 Gy drop in the mean dose to lung-avoid, and/or a 1 Gy reduction in mean dose to lung-vent
- V20 and mean dose to the anatomical lungs should be as similar as possible between the two plans

If these goals cannot be achieved, a decision will be made by the physicist and the treating physician as to whether the patient should be excluded from the trial.

Once both plans are deemed acceptable, the radiation oncologist, dosimetrist, and physicist will review and approve both plans. When the radiation oncologist reviews the two plans, machine parameters will be hidden; these parameters will be viewable by the radiation oncologist only after the randomization step (see below), and only for the clinically-selected plan. To ensure consistency, the two plans will have a V20 for the anatomically-defined lungs (lung minus IGTV) within 3% of one another. While there are no other explicit constraints for the two plans relative to one-another, in the best clinical judgment of the treating physician, neither plan should be clearly superior in terms of either target coverage or organ-at-risk (OAR) doses (excluding the anatomically-defined lungs).

The doses to other OARs must be considered clinically equivalent. Both plans will be printed and signed. The patient will then be randomized (see randomization below).

After randomization, one physicist (termed the ‘unblinded physicist’) will be responsible for receiving the randomization results for all patients in this trial. This unblinded physicist will choose the applicable plan for clinical use. This will be labelled as “Clinical Plan” and placed in the treatment system for standard quality assurance, and functional lung contours will be removed from that plan to maintain blinding. The previous two plans (*standard* and *avoidance*) will be archived under password protection. The standard treatment binder will include a printout of the clinical plan but will not contain information that would allow for unblinding.

It is generally required that an unblinding procedure be available in case of emergency. However, it is unlikely that unblinding would be required under any circumstance, even in the event of a radiation complication, as the doses delivered to normal structures will always be available in the treatment binder, wherein the treatment arm would not be apparent. In the unlikely event of an unforeseen emergency where unblinding is required, the unblinded physicist would be contacted and would confirm with the radiation oncologist that unblinding is required. That physicist would then access the password-protected plan to determine which plan was selected. The unblinded physicist will not be involved in patient care or ascertainment of outcomes.

In the event that a patient should require a repeat CT simulation for an unforeseen reason (e.g. pulmonary re-expansion, new atelectasis, rapid reduction in tumour bulk), the planning procedure above will be repeated, with both a standard plan and an avoidance plan being created. The unblinded physicist will not be involved in any of the re-planning steps up until the randomization step. Both re-plans (*standard* and *avoidance*) will need to be approved by the radiation oncologist. At this point, the unblinded physicist will select the appropriate plan according to the previous randomization. The post-randomization procedure will then continue as described below.

### Chemotherapy and treatment sequencing

Platinum-based chemotherapy will be delivered by the medical oncologist according to local standards. Radiotherapy will usually start with cycle 2 of chemotherapy. However, at the discretion of the treating physician,radiotherapy may be started with cycle 1 (urgent cases), or after more than 2 cycles (e.g. where tumour downsizing is required). Concurrent chemotherapy cannot contain taxanes or gemcitabine.

### Follow-up and assessment of efficacy

#### Quality of life (QOL) and health utilities

QOL and health-utility data will be collected using the FACT-L scale and the EQ-5D, respectively.

The primary QOL endpoint will be based on the FACT-LCS (a subset of FACT-L) which measures pulmonary QOL specifically. As a secondary endpoint, the FACT-TOI incorporates the LCS with two additional domains from FACT-L: the Physical Well Being (PWB) and Functional Well-Being (FWB) scores. Although only subsets of FACT-L are used for these two endpoints, it is generally recommended that the full FACT-L be completed during clinical trials for more robust assessment of other QOL domains.

The EQ-5D measures health utility, and is used for calculating quality-adjusted survival and cost-utility.

#### Follow-up

Patients will be assessed by the radiation oncologist at 3 months, 6 months and 1 year, then every 6 months until two years, then annually until 5 years (Table 2). A detailed history, physical examinations and CT chest and upper abdomen will be performed with each assessment. Other investigations are as follows:

**Table 2:** **Follow-up schedule**

	Before entry	Last week of radiotherapy	Month 3	Month 6	Bianually for 2 years then annually until year 5
History and Physical	X	X	X	X	X
Staging Imaging (see section 5)	X				
Pulmonary function tests and 6MWT at Robarts Research Institute	X		X		X
Baseline Bloodwork (see section 5)	X				
Pregnancy test for women of child-bearing age	X				
Toxicity and QOL Scoring (PAR, FACT-L, EQ-5D)	X	X	X	X	X (month 12 only)
Follow-up CT chest/upper abdomen			X	X	X

- Toxicity scoring and QOL scoring (FACT-L and EQ-5D): during the last week of radiotherapy (i.e. at last patient review clinical visit), and at 3-, 6- and 12-months post-treatment.
- Pulmonary Function Tests and 6MWT: 3-months and 12-months post-treatment

### Statistics and sample size calculation

#### Sample size

The primary endpoint is the QOL score on the FACT-LCS measured 3-months post-treatment. A change in the LCS score of 2-3 points is considered clinically relevant [32]. Following concurrent CRT, it is assumed that in the arm receiving standard treatment, the mean post-treatment LCS score will be 20 [33]. The study will use a two-sided, independent-sample t-test with an alpha level of 0.05 and power of 80%, and will assume a 3-month QOL non-completion rate of 10%. The standard deviation of the LCS score is estimated to be 4. In order to detect a 3-point improvement in QOL in the experimental arm (Arm 2), a total of 64 patients will be required (32 in each arm).

#### Randomization

Randomization will occur in a 1:1 ratio between Arm 1 and Arm 2 using permuted blocks. Randomization results will be communicated by the statistician to the unblinded physicist by telephone.

#### Analysis plan

Patients will be analysed in the groups to which they are assigned (intention-to-treat). An independent-sample t-test will be used to compare QOL scores at 3-months. The percentage of patients in each arm who experience a clinically significant QOL decline (3 points) will also be reported. Survival will be calculated from date of randomization using the Kaplan-Meier method with differences compared using the log-rank test. A Cox multivariable regression analysis will be used to determine baseline factors predictive of survival. For the secondary endpoints involving QOL scales, linear mixed effects models will be used.

#### Data safety monitoring committee

The data safety monitoring committee (DSMC) will consist of a statistician, an independent investigator, and a content expert. The DSMC will review toxicity outcomes on a semi-annual basis. If any grade 3-5 toxicity is reported, the patient case will be reviewed to determine if such toxicity is related to treatment. The DSMC may recommend modification or cessation of the trial if radiotherapy toxicity rates are deemed excessive (e.g. >10% grade 5 toxicity).

#### Interim analysis

The DSMC will conduct one interim analysis once 32 patients have been accrued and followed for 3 months. For this analysis, the DSMC will be blinded to the identity of each treatment arm, but QOL and OS data will be presented for each arm.

The DSMC will recommend stopping the trial if there is an OS difference that is statistically significant with a threshold of p < 0.001 using the log-rank test, based on the Haybittle-Peto stopping rule. This retains an overall alpha of 0.05.

At the Interim Analysis, the DSMC will also check the validity of the sample size calculation assumptions. The DSMC will be provided with the standard deviation of the FACT-LCS scores in each arm (while remaining blinded to the identity of each arm), and the rate of completion of the 3-month QOL forms. If these values are substantially different than estimated in the sample size calculation, the DSMC can recommend increasing or decreasing the target accrual in order to maintain statistical power.

### Institutional research ethics board (REB) approval

Western University REB Number: 104834 transferred under methods section.

## Discussion

The goal of this randomized, double-blind trial is to comprehensively evaluate the effect of functional lung avoidance using pulmonary functional imaging on both pulmonary toxicity and QOL, specifically for patients receiving concurrent CRT for locally advanced NSCLC.

To date, SPECT and 4DCT have been used in radiation treatment planning to provide functional lung information. These investigations have demonstrated the reduction of dose to healthy lung tissue [11, 16]. However, to our knowledge, they have not assessed patient QOL as one of their primary endpoints, which has demonstrated to be a powerful tool to capture pulmonary toxicity outcomes [9].

Hyperpolarized ^3^He MRI provides an alternative to SPECT and 4DCT and offers high spatial and temporal resolution of respiratory function [15]. Although ^3^He has several inherent advantages, it will not likely achieve widespread clinical use, due to cost and a limited global supply of helium. However, several alternatives appear promising and are expected to be available for widespread clinical use in the future, including ^129^Xe MRI and ^1^H Fourier Decomposition, which are currently less well-developed than ^3^He MRI and 4DCT. By establishing the benefits of ^3^He MRI functional lung avoidance and validating it against less developed methods such as ^129^Xe and ^1^H Fourier Decomposition MRI, these latter methods will allow for widespread implementation of functional lung avoidance radiotherapy.

In summary, this study will determine if ^3^He MRI-based functional lung avoidance methods will improve QOL and pulmonary toxicity in subjects with unresectable NSCLC.

## Abbreviations

4DCT: Four-dimensional computed tomography
6MWT: Six minute walk test
AE: Adverse event
AJCC: American joint committee on cancer
ATS: American thoracic society
CBC: Complete blood count
CRT: Chemoradiotherapy
CT: Computed tomography
CTCAE: Common terminology criteria for adverse events
CTV: Clinical target volume
DLCO: Diffusing capacity of carbon monoxide
DSMC: Data safety monitoring committee
ECOG: Eastern cooperative oncology group
EQ-5D: EuroQOL five dimensions questionnaire
ERS: European respiratory society
FACT: Functional assessment of cancer therapy
FACT-L: FACT-lung
FACT-LCS: FACT-lung cancer subscale
FACT-TOI: FACT-trial outcomes index
FDG: Fluorodeoxyglucose
FEV_1_: Forced expiratory volume in 1 second
FRC: Functional residual capacity
GTV: Gross tumour volume
IGTV: Internal GTV
IMRT: Intensity modulated radiation therapy
ITV: Internal target volume
MR: Magnetic resonance
MRI: MR imaging
NCI-CTC: National cancer institute common toxicity criteria
NSCLC: Non-small cell lung cancer
OAR: Organ(s) at risk
OS: Overall survival
PET: Positron emission tomography
PTV: Planning target volume
QOL: Quality of life
REB: Research ethics board
RECIST: Response evaluation criteria in solid tumours
RILI: Radiation-induced lung injury
RP: Radiation pneumonitis
RTOG: Radiation therapy oncology group
SAE: Serious adverse event
SPECT: Single photon emission computed tomography
TE: Echo time
TR: Repetition time
V20: Volume of lung receiving at least 20 Gy
VMAT: Volumetric modulated arc therapy.

## References

[1] Ferlay J, Soerjomataram I, Ervik M, Dikshit R, Eser S, Mathers C, Rebelo M, Parkin DM, Forman D, Bray F (2013). GLOBOCAN 2012 v1.0, Cancer Incidence and Mortality Worldwide: IARC CancerBase No. 11.

[2] Albain KS, Swann RS, Rusch VW, Turrisi AT, Shepherd FA, Smith C, Chen Y, Livingston RB, Feins RH, Gandara DR, Fry WA, Darling G, Johson DH, Green MR, Miller RC, Ley J, Sause WT, Cox JD (2009). Radiotherapy plus chemotherapy with or without surgical resection for stage III non-small-cell lung cancer: a phase III randomised controlled trial. Lancet.

[3] van Meerbeeck JP, Kramer GW, Van Schil PE, Legrand C, Smit EF, Schramel F, Tjan-Heijnen VC, Biesma B, Debruyne C, van Zandwijk N, Splinter TA, Giacconne G, European Organisation for Research and Treatment of Cancer-Lung Cancer Group (2007). Randomized controlled trial of resection versus radiotherapy after induction chemotherapy in stage IIIA-N2 non-small-cell lung cancer. J Natl Cancer Inst.

[4] O’Rourke N, Roque IFM, Farre Bernado N, Macbeth F (2010). Concurrent chemoradiotherapy in non-small cell lung cancer. Cochrane Database Syst Rev.

[5] Curran WJ, Paulus R, Langer CJ, Komaki R, Lee JS, Hauser S, Movsas B, Wasserman T, Rosenthal SA, Gore E, Machtay M, Sause W, Cox JD (2011). Sequential vs. concurrent chemoradiation for stage III non-small cell lung cancer: randomized phase III trial RTOG 9410. J Natl Cancer Inst.

[6] Rodrigues G, Lock M, D’Souza D, Yu E, Van Dyk J (2004). Prediction of radiation pneumonitis by dose - volume histogram parameters in lung cancer–a systematic review. Radiother Oncol.

[7] Palma DA, Senan S, Tsujino K, Barriger RB, Rengan R, Moreno M, Bradley JD, Kim TH, Ramella S, Marks LB, De Petris L, Stitt L, Rodrigues G (2011). Predicting radiation pneumonitis after chemoradiotherapy for lung cancer: an international individual patient data meta-analysis submitted, under review. Int J Radiat Oncol Biol Phys.

[8] Tsujino K, Hirota S, Endo M, Obayashi K, Kotani Y, Satouchi M, Kado T, Takada Y (2003). Predictive value of dose-volume histogram parameters for predicting radiation pneumonitis after concurrent chemoradiation for lung cancer. Int J Radiat Oncol Biol Phys.

[9] Movsas B, Hu C, Sloan J, Bradley JD, Kavadi VS, Narayan S, Robinson C, Johnson DW, Paulus R, Choy H (2013). Quality of life (QOL) analysis of the randomized radiation (RT) dose-escalation NSCLC trial (RTOG 0617): the rest of the story. Int J Radiat Oncol Biol Phys.

[10] Shioyama Y, Jang SY, Liu HH, Guerrero T, Wang X, Gayed IW, Erwin WD, Liao Z, Chang JY, Jeter M, Yaremko BP, Borghero YO, Cox JD, Komaki R, Mohan R (2007). Preserving functional lung using perfusion imaging and intensity-modulated radiation therapy for advanced-stage non-small cell lung cancer. Int J Radiat Oncol Biol Phys.

[11] Yaremko BP, Guerrero TM, Noyola-Martinez J, Guerra R, Lege DG, Nguyen LT, Balter PA, Cox JD, Komaki R (2007). Reduction of normal lung irradiation in locally advanced non-small-cell lung cancer patients, using ventilation images for functional avoidance. Int J Radiat Oncol Biol Phys.

[12] Ding K, Bayouth JE, Buatti JM, Christensen GE, Reinhardt JM (2010). 4DCT-based measurement of changes in pulmonary function following a course of radiation therapy. Med Phys.

[13] Vinogradskiy YY, Castillo R, Castillo E, Chandler A, Martel MK, Guerrero T (2012). Use of weekly 4DCT-based ventilation maps to quantify changes in lung function for patients undergoing radiation therapy. Med Phys.

[14] Hodge CW, Tome WA, Fain SB, Bentzen SM, Mehta MP (2010). On the use of hyperpolarized helium MRI for conformal avoidance lung radiotherapy. Med Dosim.

[15] Ireland RH, Bragg CM, McJury M, Woodhouse N, Fichele S, van Beek EJ, Wild JM, Hatton MQ (2007). Feasibility of image registration and intensity-modulated radiotherapy planning with hyperpolarized helium-3 magnetic resonance imaging for non-small-cell lung cancer. Int J Radiat Oncol Biol Phys.

[16] Lavrenkov K, Singh S, Christian JA, Partridge M, Nioutsikou E, Cook G, Bedford JL, Brada M (2009). Effective avoidance of a functional spect-perfused lung using intensity modulated radiotherapy (IMRT) for non-small cell lung cancer (NSCLC): an update of a planning study. Radiother Oncol.

[17] Jogi J, Jonson B, Ekberg M, Bajc M (2010). Ventilation-perfusion SPECT with 99mTc-DTPA versus Technegas: a head-to-head study in obstructive and nonobstructive disease. J Nucl Med.

[18] Mathew L, Gaede S, Wheatley A, Etemad-Rezai R, Rodrigues GB, Parraga G (2010). Detection of longitudinal lung structural and functional changes after diagnosis of radiation-induced lung injury using hyperpolarized 3He magnetic resonance imaging. Med Phys.

[19] Fain S, Schiebler ML, McCormack DG, Parraga G (2010). Imaging of lung function using hyperpolarized helium-3 magnetic resonance imaging: Review of current and emerging translational methods and applications. J Magn Reson Imaging.

[20] Patz S, Muradian I, Hrovat MI, Ruset IC, Topulos G, Covrig SD, Frederick E, Hatabu H, Hersman FW, Butler JP (2008). Human pulmonary imaging and spectroscopy with hyperpolarized 129Xe at 0.2T. Acad Radiol.

[21] Bauman G, Puderbach M, Deimling M, Jellus V, Chefd’hotel C, Dinkel J, Hintze C, Kauczor HU, Schad LR (2009). Non-contrast-enhanced perfusion and ventilation assessment of the human lung by means of fourier decomposition in proton MRI. Magn Reson Med.

[22] Mathew L, Wheatley A, Castillo R, Castillo E, Rodrigues G, Guerrero T, Parraga G (2012). Hyperpolarized 3He magnetic resonance imaging: comparison with four-dimensional x-ray computed tomography imaging in lung cancer. Acad Radiol.

[23] Miller MR, Crapo R, Hankinson J, Brusasco V, Burgos F, Casaburi R, Coates A, Enright P, van der Grinten CPM, Gustafsson P, Jensen R, Johnson DC, MacIntyre N, McKay R, Navajas D, Pedersen OF, Pellegrino R, Viegi G, Wanger J, ATS/ERS Task Force (2005). General considerations for lung function testing. Eur Respir J.

[24] Miller MR, Hankinson J, Brusasco V, Burgos F, Casaburi R, Coates A, Crapo R, Enright P, van der Grinten CPM, Gustafsson P, Jensen R, Johnson DC, MacIntyre N, McKay R, Navajas D, Pedersen OF, Pellegrino R, Viegi G, Wanger J, ATS/ERS Task Force (2005). Standardisation of spirometry. Eur Respir J.

[25] Pellegrino R, Viegi G, Brusasco V, Crapo RO, Burgos F, Casaburi R, Coates A, van der Grinten CPM, Gustafsson P, Hankinson J, Jensen R, Johnson DC, MacIntyre N, McKay R, Miller MR, Navajas D, Pedersen OF, Wanger J (2005). Interpretative strategies for lung function tests. Eur Respir J.

[26] Wanger J, Clausen JL, Coates A, Pedersen OF, Brusasco V, Burgos F, Casaburi R, Crapo R, Enright P, van der Grinten CPM, Gustafsson P, Hankinson J, Jensen R, Johnson D, MacIntyre N, McKay R, Miller MR, Navajas D, Pellegrino R, Viegi G (2005). Standardisation of the measurement of lung volumes. Eur Respir J.

[27] Macintyre N, Crapo RO, Viegi G, Johnson DC, van der Grinten CPM, Brusasco V, Burgos F, Casaburi R, Coates A, Enright P, Gustafsson P, Hankinson J, Jensen R, McKay R, Miller MR, Navajas D, Pedersen OF, Pellegrino R, Wanger J (2005). Standardisation of the single-breath determination of carbon monoxide uptake in the lung. Eur Respir J.

[28] AmericanThoracicSociety (2002). ATS statement: guidelines for the six-minute walk test. Am J Respir Crit Care Med.

[29] Siegl P, Schultz K (1984). The Borg scale as an instrument for the detection of subjectively experienced stress in industrial medicine laboratory and field studies. Z Gesamte Hyg.

[30] Parraga G, Ouriadov A, Evans A, McKay S, Lam WW, Fenster A, Etemad-Rezai R, McCormack D, Santyr G (2007). Hyperpolarized 3He ventilation defects and apparent diffusion coefficients in chronic obstructive pulmonary disease: preliminary results at 3.0 Tesla. Invest Radiol.

[31] *Protocol: RTOG 0617*. http://www.rtog.org/ClinicalTrials/ProtocolTable/StudyDetails.aspx?study=0617

[32] Cella D, Eton DT, Fairclough DL, Bonomi P, Heyes AE, Silberman C, Wolf MK, Johnson DH (2002). What is a clinically meaningful change on the functional assessment of cancer therapy-lung (FACT-L) questionnaire? Results from eastern cooperative oncology group (ECOG) study 5592. J Clin Epidemiol.

[33] Auchter RM, Scholtens D, Adak S, Wagner H, Cella DF, Mehta MP (2001). Quality of life assessment in advanced non-small-cell lung cancer patients undergoing an accelerated radiotherapy regimen: report of ECOG study 4593. Eastern cooperative oncology group. Int J Radiat Oncol Biol Phys.

[CR34] The pre-publication history for this paper can be accessed here: http://www.biomedcentral.com/1471-2407/14/934/prepub

